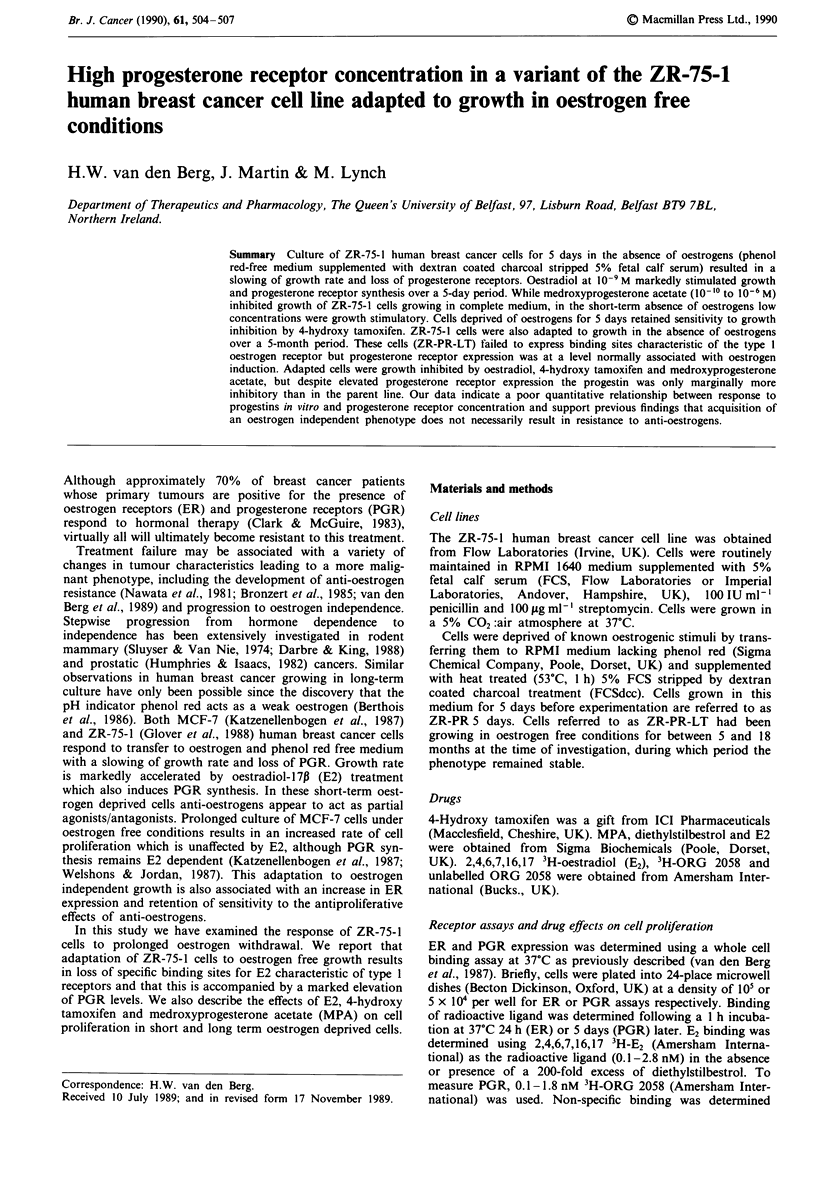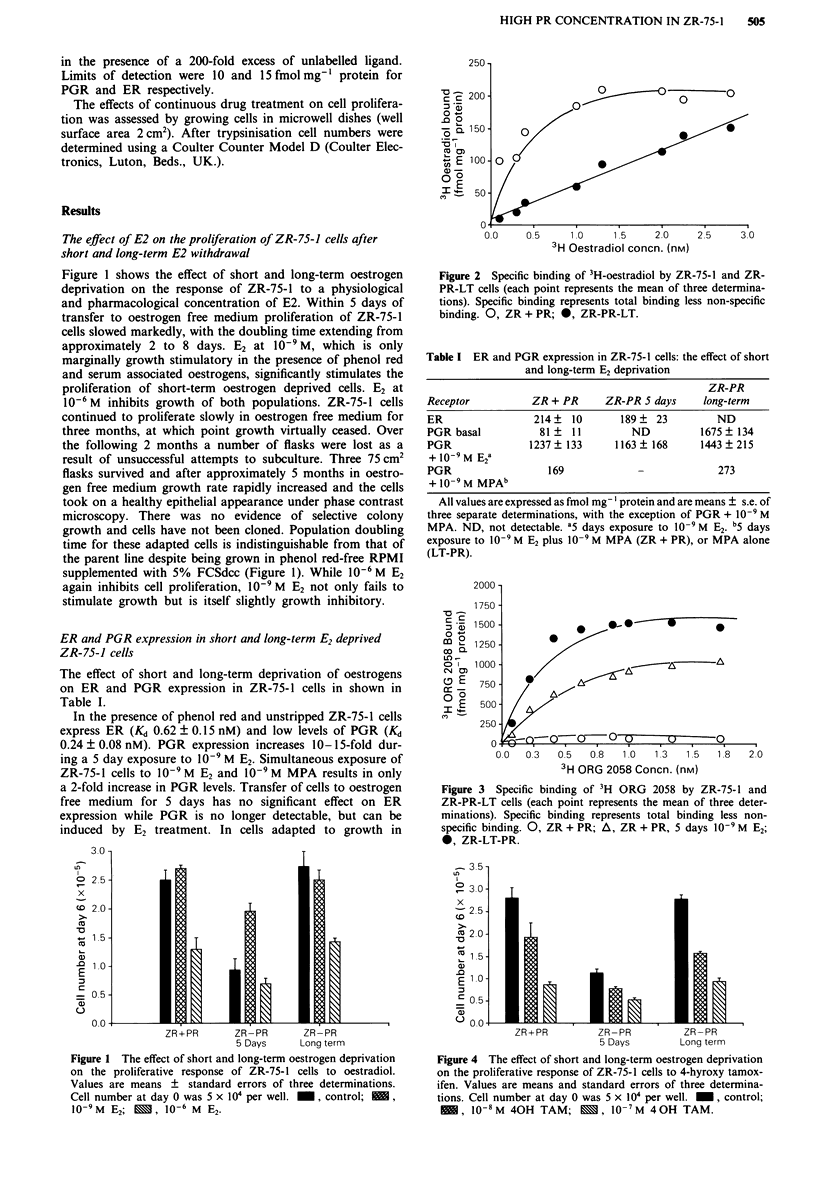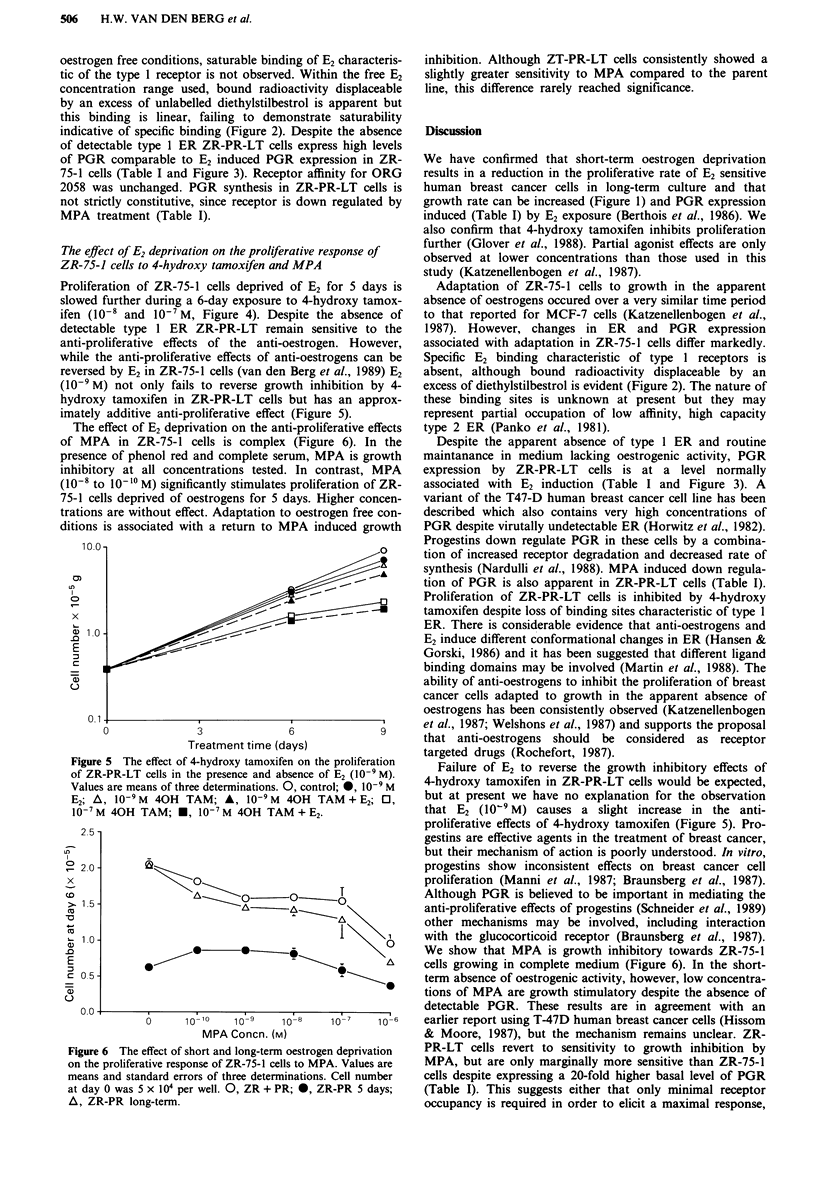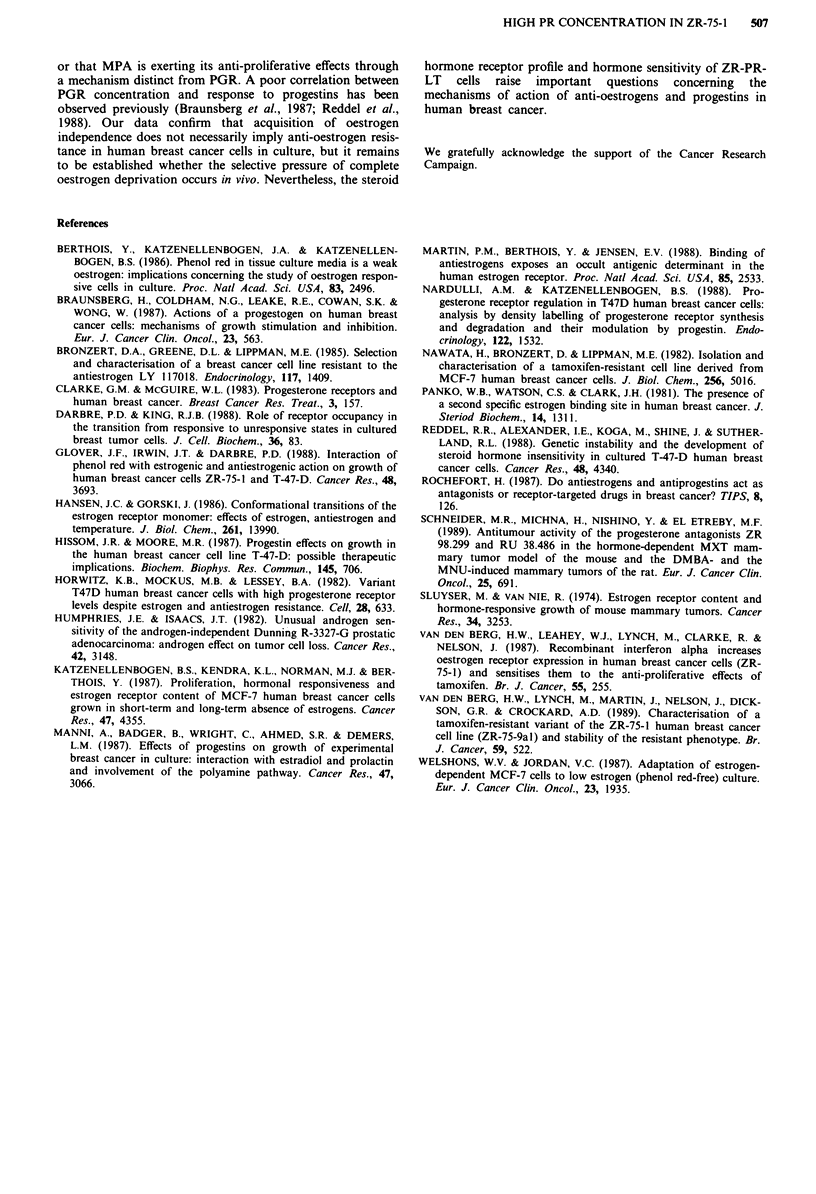# High progesterone receptor concentration in a variant of the ZR-75-1 human breast cancer cell line adapted to growth in oestrogen free conditions.

**DOI:** 10.1038/bjc.1990.114

**Published:** 1990-04

**Authors:** H. W. van den Berg, J. Martin, M. Lynch

**Affiliations:** Department of Therapeutics and Pharmacology, Queen's University of Belfast, Northern Ireland.

## Abstract

Culture of ZR-75-1 human breast cancer cells for 5 days in the absence of oestrogens (phenol red-free medium supplemented with dextran coated charcoal stripped 5% fetal calf serum) resulted in a slowing of growth rate and loss of progesterone receptors. Oestradiol at 10(-9) M markedly stimulated growth and progesterone receptor synthesis over a 5-day period. While medroxyprogesterone acetate (10(-10) to 10(-6) M) inhibited growth of ZR-75-1 cells growing in complete medium, in the short-term absence of oestrogens low concentrations were growth stimulatory. Cells deprived of oestrogens for 5 days retained sensitivity to growth inhibition by 4-hydroxy tamoxifen. ZR-75-1 cells were also adapted to growth in the absence of oestrogens over a 5-month period. These cells (ZR-PR-LT) failed to express binding sites characteristic of the type 1 oestrogen receptor but progesterone receptor expression was at a level normally associated with oestrogen induction. Adapted cells were growth inhibited by oestradiol, 4-hydroxy tamoxifen and medroxyprogesterone acetate, but despite elevated progesterone receptor expression the progestin was only marginally more inhibitory than in the parent line. Our data indicate a poor quantitative relationship between response to progestins in vitro and progesterone receptor concentration and support previous findings that acquisition of an oestrogen independent phenotype does not necessarily result in resistance to anti-oestrogens.


Br. J. Cancer (1990), 61, 504–507 © Macmillan Press Ltd., 1990

### High progesterone receptor concentration in a variant of the ZR-75-1 human breast cancer cell line adapted to growth in oestrogen free conditions

H.W. van den Berg, J. Martin & M. Lynch

*Department of Therapeutics and Pharmacology, The Queen's University of Belfast, 97, Lisburn Road, Belfast BT9 7BL, Northern Ireland.*

**Summary** Culture of ZR-75-1 human breast cancer cells for 5 days in the absence of oestrogens (phenol red-free medium supplemented with dextran coated charcoal stripped 5% fetal calf serum) resulted in a slowing of growth rate and loss of progesterone receptors. Oestradiol at $10^{-9}$ M markedly stimulated growth and progesterone receptor synthesis over a 5-day period. While medroxyprogesterone acetate ($10^{-10}$ to $10^{-6}$ M) inhibited growth of ZR-75-1 cells growing in complete medium, in the short-term absence of oestrogens low concentrations were growth stimulatory. Cells deprived of oestrogens for 5 days retained sensitivity to growth inhibition by 4-hydroxy tamoxifen. ZR-75-1 cells were also adapted to growth in the absence of oestrogens over a 5-month period. These cells (ZR-PR-LT) failed to express binding sites characteristic of the type 1 oestrogen receptor but progesterone receptor expression was at a level normally associated with oestrogen induction. Adapted cells were growth inhibited by oestradiol, 4-hydroxy tamoxifen and medroxyprogesterone acetate, but despite elevated progesterone receptor expression the progestin was only marginally more inhibitory than in the parent line. Our data indicate a poor quantitative relationship between response to progestins *in vitro* and progesterone receptor concentration and support previous findings that acquisition of an oestrogen independent phenotype does not necessarily result in resistance to anti-oestrogens.

Although approximately 70% of breast cancer patients whose primary tumours are positive for the presence of oestrogen receptors (ER) and progesterone receptors (PGR) respond to hormonal therapy (Clark & McGuire, 1983), virtually all will ultimately become resistant to this treatment.

Treatment failure may be associated with a variety of changes in tumour characteristics leading to a more malignant phenotype, including the development of anti-oestrogen resistance (Nawata *et al.*, 1981; Bronzert *et al.*, 1985; van den Berg *et al.*, 1989) and progression to oestrogen independence. Stepwise progression from hormone dependence to independence has been extensively investigated in rodent mammary (Sluyser & Van Nie, 1974; Darbre & King, 1988) and prostatic (Humphries & Isaacs, 1982) cancers. Similar observations in human breast cancer growing in long-term culture have only been possible since the discovery that the pH indicator phenol red acts as a weak oestrogen (Berthois *et al.*, 1986). Both MCF-7 (Katzenellenbogen *et al.*, 1987) and ZR-75-1 (Glover *et al.*, 1988) human breast cancer cells respond to transfer to oestrogen and phenol red free medium with a slowing of growth rate and loss of PGR. Growth rate is markedly accelerated by oestradiol-17$\beta$ (E2) treatment which also induces PGR synthesis. In these short-term oestrogen deprived cells anti-oestrogens appear to act as partial agonists/antagonists. Prolonged culture of MCF-7 cells under oestrogen free conditions results in an increased rate of cell proliferation which is unaffected by E2, although PGR synthesis remains E2 dependent (Katzenellenbogen *et al.*, 1987; Welshons & Jordan, 1987). This adaptation to oestrogen independent growth is also associated with an increase in ER expression and retention of sensitivity to the antiproliferative effects of anti-oestrogens.

In this study we have examined the response of ZR-75-1 cells to prolonged oestrogen withdrawal. We report that adaptation of ZR-75-1 cells to oestrogen free growth results in loss of specific binding sites for E2 characteristic of type 1 receptors and that this is accompanied by a marked elevation of PGR levels. We also describe the effects of E2, 4-hydroxy tamoxifen and medroxyprogesterone acetate (MPA) on cell proliferation in short and long term oestrogen deprived cells.

Correspondence: H.W. van den Berg.
Received 10 July 1989; and in revised form 17 November 1989.

### Materials and methods

### *Cell lines*

The ZR-75-1 human breast cancer cell line was obtained from Flow Laboratories (Irvine, UK). Cells were routinely maintained in RPMI 1640 medium supplemented with 5% fetal calf serum (FCS, Flow Laboratories or Imperial Laboratories, Andover, Hampshire, UK), 100 IU $ml^{-1}$ penicillin and 100 $\mu g\ ml^{-1}$ streptomycin. Cells were grown in a 5% $CO_2$:air atmosphere at 37°C.

Cells were deprived of known oestrogenic stimuli by transferring them to RPMI medium lacking phenol red (Sigma Chemical Company, Poole, Dorset, UK) and supplemented with heat treated (53°C, 1 h) 5% FCS stripped by dextran coated charcoal treatment (FCSdcc). Cells grown in this medium for 5 days before experimentation are referred to as ZR-PR 5 days. Cells referred to as ZR-PR-LT had been growing in oestrogen free conditions for between 5 and 18 months at the time of investigation, during which period the phenotype remained stable.

### *Drugs*

4-Hydroxy tamoxifen was a gift from ICI Pharmaceuticals (Macclesfield, Cheshire, UK). MPA, diethylstilbestrol and E2 were obtained from Sigma Biochemicals (Poole, Dorset, UK). 2,4,6,7,16,17 $^3$H-oestradiol ($E_2$), $^3$H-ORG 2058 and unlabelled ORG 2058 were obtained from Amersham International (Bucks., UK).

### *Receptor assays and drug effects on cell proliferation*

ER and PGR expression was determined using a whole cell binding assay at 37°C as previously described (van den Berg *et al.*, 1987). Briefly, cells were plated into 24-place microwell dishes (Becton Dickinson, Oxford, UK) at a density of $10^5$ or $5 \times 10^4$ per well for ER or PGR assays respectively. Binding of radioactive ligand was determined following a 1 h incubation at 37°C 24 h (ER) or 5 days (PGR) later. $E_2$ binding was determined using 2,4,6,7,16,17 $^3$H-$E_2$ (Amersham International) as the radioactive ligand (0.1–2.8 nM) in the absence or presence of a 200-fold excess of diethylstilbestrol. To measure PGR, 0.1–1.8 nM $^3$H-ORG 2058 (Amersham International) was used. Non-specific binding was determined in the presence of a 200-fold excess of unlabelled ligand. Limits of detection were 10 and 15 fmol $mg^{-1}$ protein for PGR and ER respectively.

The effects of continuous drug treatment on cell proliferation was assessed by growing cells in microwell dishes (well surface area 2 $cm^2$). After trypsinisation cell numbers were determined using a Coulter Counter Model D (Coulter Electronics, Luton, Beds., UK.).

### Results

### *The effect of E2 on the proliferation of ZR-75-1 cells after short and long-term E2 withdrawal*

Figure 1 shows the effect of short and long-term oestrogen deprivation on the response of ZR-75-1 to a physiological and pharmacological concentration of E2. Within 5 days of transfer to oestrogen free medium proliferation of ZR-75-1 cells slowed markedly, with the doubling time extending from approximately 2 to 8 days. $E_2$ at $10^{-9}$ M, which is only marginally growth stimulatory in the presence of phenol red and serum associated oestrogens, significantly stimulates the proliferation of short-term oestrogen deprived cells. $E_2$ at $10^{-6}$ M inhibits growth of both populations. ZR-75-1 cells continued to proliferate slowly in oestrogen free medium for three months, at which point growth virtually ceased. Over the following 2 months a number of flasks were lost as a result of unsuccessful attempts to subculture. Three 75 $cm^2$ flasks survived and after approximately 5 months in oestrogen free medium growth rate rapidly increased and the cells took on a healthy epithelial appearance under phase contrast microscopy. There was no evidence of selective colony growth and cells have not been cloned. Population doubling time for these adapted cells is indistinguishable from that of the parent line despite being grown in phenol red-free RPMI supplemented with 5% FCSdcc (Figure 1). While $10^{-6}$ M $E_2$ again inhibits cell proliferation, $10^{-9}$ M $E_2$ not only fails to stimulate growth but is itself slightly growth inhibitory.

### *ER and PGR expression in short and long-term $E_2$ deprived ZR-75-1 cells*

The effect of short and long-term deprivation of oestrogens on ER and PGR expression in ZR-75-1 cells in shown in Table I.

In the presence of phenol red and unstripped ZR-75-1 cells express ER ($K_d$ 0.62 ± 0.15 nM) and low levels of PGR ($K_d$ 0.24 ± 0.08 nM). PGR expression increases 10–15-fold during a 5 day exposure to $10^{-9}$ M $E_2$. Simultaneous exposure of ZR-75-1 cells to $10^{-9}$ M $E_2$ and $10^{-9}$ M MPA results in only a 2-fold increase in PGR levels. Transfer of cells to oestrogen free medium for 5 days has no significant effect on ER expression while PGR is no longer detectable, but can be induced by $E_2$ treatment. In cells adapted to growth in

**Figure 1** The effect of short and long-term oestrogen deprivation on the proliferative response of ZR-75-1 cells to oestradiol. Values are means ± standard errors of three determinations. Cell number at day 0 was 5 × $10^4$ per well. ■, control; ▩, $10^{-9}$ M $E_2$; ▧, $10^{-6}$ M $E_2$.

**Figure 2** Specific binding of $^3$H-oestradiol by ZR-75-1 and ZR-PR-LT cells (each point represents the mean of three determinations). Specific binding represents total binding less non-specific binding. ○, ZR + PR; ●, ZR-PR-LT.

**Table I** ER and PGR expression in ZR-75-1 cells: the effect of short and long-term $E_2$ deprivation

| *Receptor* | *ZR + PR* | *ZR-PR 5 days* | *ZR-PR long-term* |
|---|---|---|---|
| ER | 214 ± 10 | 189 ± 23 | ND |
| PGR basal | 81 ± 11 | ND | 1675 ± 134 |
| PGR + $10^{-9}$ M $E_2$[a] | 1237 ± 133 | 1163 ± 168 | 1443 ± 215 |
| PGR + $10^{-9}$ M MPA[b] | 169 | – | 273 |

All values are expressed as fmol $mg^{-1}$ protein and are means ± s.e. of three separate determinations, with the exception of PGR + $10^{-9}$ M MPA. ND, not detectable. [a]5 days exposure to $10^{-9}$ M $E_2$. [b]5 days exposure to $10^{-9}$ M $E_2$ plus $10^{-9}$ M MPA (ZR + PR), or MPA alone (LT-PR).

**Figure 3** Specific binding of $^3$H ORG 2058 by ZR-75-1 and ZR-PR-LT cells (each point represents the mean of three determinations). Specific binding represents total binding less non-specific binding. ○, ZR + PR; △, ZR + PR, 5 days $10^{-9}$ M $E_2$; ●, ZR-LT-PR.

**Figure 4** The effect of short and long-term oestrogen deprivation on the proliferative response of ZR-75-1 cells to 4-hyroxy tamoxifen. Values are means and standard errors of three determinations. Cell number at day 0 was 5 × $10^4$ per well. ■, control; ▩, $10^{-8}$ M 4OH TAM; ▧, $10^{-7}$ M 4OH TAM.

oestrogen free conditions, saturable binding of $E_2$ characteristic of the type 1 receptor is not observed. Within the free $E_2$ concentration range used, bound radioactivity displaceable by an excess of unlabelled diethylstilbestrol is apparent but this binding is linear, failing to demonstrate saturability indicative of specific binding (Figure 2). Despite the absence of detectable type 1 ER ZR-PR-LT cells express high levels of PGR comparable to $E_2$ induced PGR expression in ZR-75-1 cells (Table I and Figure 3). Receptor affinity for ORG 2058 was unchanged. PGR synthesis in ZR-PR-LT cells is not strictly constitutive, since receptor is down regulated by MPA treatment (Table I).

### *The effect of $E_2$ deprivation on the proliferative response of ZR-75-1 cells to 4-hydroxy tamoxifen and MPA*

Proliferation of ZR-75-1 cells deprived of $E_2$ for 5 days is slowed further during a 6-day exposure to 4-hydroxy tamoxifen ($10^{-8}$ and $10^{-7}$ M, Figure 4). Despite the absence of detectable type 1 ER ZR-PR-LT remain sensitive to the anti-proliferative effects of the anti-oestrogen. However, while the anti-proliferative effects of anti-oestrogens can be reversed by $E_2$ in ZR-75-1 cells (van den Berg et al., 1989) $E_2$ ($10^{-9}$ M) not only fails to reverse growth inhibition by 4-hydroxy tamoxifen in ZR-PR-LT cells but has an approximately additive anti-proliferative effect (Figure 5).

The effect of $E_2$ deprivation on the anti-proliferative effects of MPA in ZR-75-1 cells is complex (Figure 6). In the presence of phenol red and complete serum, MPA is growth inhibitory at all concentrations tested. In contrast, MPA ($10^{-8}$ to $10^{-10}$ M) significantly stimulates proliferation of ZR-75-1 cells deprived of oestrogens for 5 days. Higher concentrations are without effect. Adaptation to oestrogen free conditions is associated with a return to MPA induced growth inhibition. Although ZT-PR-LT cells consistently showed a slightly greater sensitivity to MPA compared to the parent line, this difference rarely reached significance.

**Figure 5** The effect of 4-hydroxy tamoxifen on the proliferation of ZR-PR-LT cells in the presence and absence of $E_2$ ($10^{-9}$ M). Values are means of three determinations. O, control; ●, $10^{-9}$ M $E_2$; Δ, $10^{-9}$ M 4OH TAM; ▲, $10^{-9}$ M 4OH TAM + $E_2$; □, $10^{-7}$ M 4OH TAM; ■, $10^{-7}$ M 4OH TAM + $E_2$.

**Figure 6** The effect of short and long-term oestrogen deprivation on the proliferative response of ZR-75-1 cells to MPA. Values are means and standard errors of three determinations. Cell number at day 0 was $5 \times 10^4$ per well. O, ZR + PR; ●, ZR-PR 5 days; Δ, ZR-PR long-term.

### Discussion

We have confirmed that short-term oestrogen deprivation results in a reduction in the proliferative rate of $E_2$ sensitive human breast cancer cells in long-term culture and that growth rate can be increased (Figure 1) and PGR expression induced (Table I) by $E_2$ exposure (Berthois et al., 1986). We also confirm that 4-hydroxy tamoxifen inhibits proliferation further (Glover et al., 1988). Partial agonist effects are only observed at lower concentrations than those used in this study (Katzenellenbogen et al., 1987).

Adaptation of ZR-75-1 cells to growth in the apparent absence of oestrogens occured over a very similar time period to that reported for MCF-7 cells (Katzenellenbogen et al., 1987). However, changes in ER and PGR expression associated with adaptation in ZR-75-1 cells differ markedly. Specific $E_2$ binding characteristic of type 1 receptors is absent, although bound radioactivity displaceable by an excess of diethylstilbestrol is evident (Figure 2). The nature of these binding sites is unknown at present but they may represent partial occupation of low affinity, high capacity type 2 ER (Panko et al., 1981).

Despite the apparent absence of type 1 ER and routine maintanance in medium lacking oestrogenic activity, PGR expression by ZR-PR-LT cells is at a level normally associated with $E_2$ induction (Table I and Figure 3). A variant of the T47-D human breast cancer cell line has been described which also contains very high concentrations of PGR despite virutally undetectable ER (Horwitz et al., 1982). Progestins down regulate PGR in these cells by a combination of increased receptor degradation and decreased rate of synthesis (Nardulli et al., 1988). MPA induced down regulation of PGR is also apparent in ZR-PR-LT cells (Table I). Proliferation of ZR-PR-LT cells is inhibited by 4-hydroxy tamoxifen despite loss of binding sites characteristic of type 1 ER. There is considerable evidence that anti-oestrogens and $E_2$ induce different conformational changes in ER (Hansen & Gorski, 1986) and it has been suggested that different ligand binding domains may be involved (Martin et al., 1988). The ability of anti-oestrogens to inhibit the proliferation of breast cancer cells adapted to growth in the apparent absence of oestrogens has been consistently observed (Katzenellenbogen et al., 1987; Welshons et al., 1987) and supports the proposal that anti-oestrogens should be considered as receptor targeted drugs (Rochefort, 1987).

Failure of $E_2$ to reverse the growth inhibitory effects of 4-hydroxy tamoxifen in ZR-PR-LT cells would be expected, but at present we have no explanation for the observation that $E_2$ ($10^{-9}$ M) causes a slight increase in the anti-proliferative effects of 4-hydroxy tamoxifen (Figure 5). Progestins are effective agents in the treatment of breast cancer, but their mechanism of action is poorly understood. *In vitro*, progestins show inconsistent effects on breast cancer cell proliferation (Manni et al., 1987; Braunsberg et al., 1987). Although PGR is believed to be important in mediating the anti-proliferative effects of progestins (Schneider et al., 1989) other mechanisms may be involved, including interaction with the glucocorticoid receptor (Braunsberg et al., 1987). We show that MPA is growth inhibitory towards ZR-75-1 cells growing in complete medium (Figure 6). In the short-term absence of oestrogenic activity, however, low concentrations of MPA are growth stimulatory despite the absence of detectable PGR. These results are in agreement with an earlier report using T-47D human breast cancer cells (Hissom & Moore, 1987), but the mechanism remains unclear. ZR-PR-LT cells revert to sensitivity to growth inhibition by MPA, but are only marginally more sensitive than ZR-75-1 cells despite expressing a 20-fold higher basal level of PGR (Table I). This suggests either that only minimal receptor occupancy is required in order to elicit a maximal response, or that MPA is exerting its anti-proliferative effects through a mechanism distinct from PGR. A poor correlation between PGR concentration and response to progestins has been observed previously (Braunsberg *et al.*, 1987; Reddel *et al.*, 1988). Our data confirm that acquisition of oestrogen independence does not necessarily imply anti-oestrogen resistance in human breast cancer cells in culture, but it remains to be established whether the selective pressure of complete oestrogen deprivation occurs *in vivo*. Nevertheless, the steroid hormone receptor profile and hormone sensitivity of ZR-PR-LT cells raise important questions concerning the mechanisms of action of anti-oestrogens and progestins in human breast cancer.

We gratefully acknowledge the support of the Cancer Research Campaign.

### References

BERTHOIS, Y., KATZENELLENBOGEN, J.A. & KATZENELLENBOGEN, B.S. (1986). Phenol red in tissue culture media is a weak oestrogen: implications concerning the study of oestrogen responsive cells in culture. *Proc. Natl Acad. Sci. USA*, **83**, 2496.

BRAUNSBERG, H., COLDHAM, N.G., LEAKE, R.E., COWAN, S.K. & WONG, W. (1987). Actions of a progestogen on human breast cancer cells: mechanisms of growth stimulation and inhibition. *Eur. J. Cancer Clin. Oncol.*, **23**, 563.

BRONZERT, D.A., GREENE, D.L. & LIPPMAN, M.E. (1985). Selection and characterisation of a breast cancer cell line resistant to the antiestrogen LY 117018. *Endocrinology*, **117**, 1409.

CLARKE, G.M. & MCGUIRE, W.L. (1983). Progesterone receptors and human breast cancer. *Breast Cancer Res. Treat.*, **3**, 157.

DARBRE, P.D. & KING, R.J.B. (1988). Role of receptor occupancy in the transition from responsive to unresponsive states in cultured breast tumor cells. *J. Cell. Biochem.*, **36**, 83.

GLOVER, J.F., IRWIN, J.T. & DARBRE, P.D. (1988). Interaction of phenol red with estrogenic and antiestrogenic action on growth of human breast cancer cells ZR-75-1 and T-47-D. *Cancer Res.*, **48**, 3693.

HANSEN, J.C. & GORSKI, J. (1986). Conformational transitions of the estrogen receptor monomer: effects of estrogen, antiestrogen and temperature. *J. Biol. Chem.*, **261**, 13990.

HISSOM, J.R. & MOORE, M.R. (1987). Progestin effects on growth in the human breast cancer cell line T-47-D: possible therapeutic implications. *Biochem. Biophys. Res. Commun.*, **145**, 706.

HORWITZ, K.B., MOCKUS, M.B. & LESSEY, B.A. (1982). Variant T47D human breast cancer cells with high progesterone receptor levels despite estrogen and antiestrogen resistance. *Cell*, **28**, 633.

HUMPHRIES, J.E. & ISAACS, J.T. (1982). Unusual androgen sensitivity of the androgen-independent Dunning R-3327-G prostatic adenocarcinoma: androgen effect on tumor cell loss. *Cancer Res.*, **42**, 3148.

KATZENELLENBOGEN, B.S., KENDRA, K.L., NORMAN, M.J. & BERTHOIS, Y. (1987). Proliferation, hormonal responsiveness and estrogen receptor content of MCF-7 human breast cancer cells grown in short-term and long-term absence of estrogens. *Cancer Res.*, **47**, 4355.

MANNI, A., BADGER, B., WRIGHT, C., AHMED, S.R. & DEMERS, L.M. (1987). Effects of progestins on growth of experimental breast cancer in culture: interaction with estradiol and prolactin and involvement of the polyamine pathway. *Cancer Res.*, **47**, 3066.

MARTIN, P.M., BERTHOIS, Y. & JENSEN, E.V. (1988). Binding of antiestrogens exposes an occult antigenic determinant in the human estrogen receptor. *Proc. Natl Acad. Sci. USA*, **85**, 2533.

NARDULLI, A.M. & KATZENELLENBOGEN, B.S. (1988). Progesterone receptor regulation in T47D human breast cancer cells: analysis by density labelling of progesterone receptor synthesis and degradation and their modulation by progestin. *Endocrinology*, **122**, 1532.

NAWATA, H., BRONZERT, D. & LIPPMAN, M.E. (1982). Isolation and characterisation of a tamoxifen-resistant cell line derived from MCF-7 human breast cancer cells. *J. Biol. Chem.*, **256**, 5016.

PANKO, W.B., WATSON, C.S. & CLARK, J.H. (1981). The presence of a second specific estrogen binding site in human breast cancer. *J. Steriod Biochem.*, **14**, 1311.

REDDEL, R.R., ALEXANDER, I.E., KOGA, M., SHINE, J. & SUTHERLAND, R.L. (1988). Genetic instability and the development of steroid hormone insensitivity in cultured T-47-D human breast cancer cells. *Cancer Res.*, **48**, 4340.

ROCHEFORT, H. (1987). Do antiestrogens and antiprogestins act as antagonists or receptor-targeted drugs in breast cancer? *TIPS*, **8**, 126.

SCHNEIDER, M.R., MICHNA, H., NISHINO, Y. & EL ETREBY, M.F. (1989). Antitumour activity of the progesterone antagonists ZR 98.299 and RU 38.486 in the hormone-dependent MXT mammary tumor model of the mouse and the DMBA- and the MNU-induced mammary tumors of the rat. *Eur. J. Cancer Clin. Oncol.*, **25**, 691.

SLUYSER, M. & VAN NIE, R. (1974). Estrogen receptor content and hormone-responsive growth of mouse mammary tumors. *Cancer Res.*, **34**, 3253.

VAN DEN BERG, H.W., LEAHEY, W.J., LYNCH, M., CLARKE, R. & NELSON, J. (1987). Recombinant interferon alpha increases oestrogen receptor expression in human breast cancer cells (ZR-75-1) and sensitises them to the anti-proliferative effects of tamoxifen. *Br. J. Cancer*, **55**, 255.

VAN DEN BERG, H.W., LYNCH, M., MARTIN, J., NELSON, J., DICKSON, G.R. & CROCKARD, A.D. (1989). Characterisation of a tamoxifen-resistant variant of the ZR-75-1 human breast cancer cell line (ZR-75-9a1) and stability of the resistant phenotype. *Br. J. Cancer*, **59**, 522.

WELSHONS, W.V. & JORDAN, V.C. (1987). Adaptation of estrogen-dependent MCF-7 cells to low estrogen (phenol red-free) culture. *Eur. J. Cancer Clin. Oncol.*, **23**, 1935.